# Prevalence of overweight/obesity and associated factors among under-five children in Ethiopia: A multilevel analysis of nationally representative sample

**DOI:** 10.3389/fpubh.2022.881296

**Published:** 2022-09-09

**Authors:** Mathewos Alemu Gebremichael, Melkamu Merid Mengesha, Samuel Hailegebreal, Hanan Abdulkadir, Asrat Arja, Biruk Bogale Wolde

**Affiliations:** ^1^Department of Public Health, College of Health Sciences, Bonga University, Bonga, Ethiopia; ^2^School of Public Health, College of Medicine and Health Sciences, Arba Minch University, Arba Minch, Ethiopia; ^3^Department of Data Repository and Governance, National Data Management Centre for Health, Ethiopian Public Health Institute, Addis Ababa, Ethiopia; ^4^School of Public Health, College of Medicine and Health Sciences, Mizan Tepi University, Mizan Aman, Ethiopia

**Keywords:** under-five children, overweight/obesity, prevalence, Ethiopia, factors

## Abstract

**Background:**

Childhood overweight and obesity are emerging public health challenges of the twety-first century. There was a 24% increase in the number of overweight children under the age of 5 years in low-income countries. Despite the significant risk of childhood overweight/obesity for non-communicable diseases, premature death, disability, and reproductive disorders in their adult life, little attention has been given. Therefore, we aimed to assess the prevalence of overweight/obesity and associated factors among under-five children.

**Methods:**

This study was conducted using data from a nationally representative sample of the 2019 Ethiopia Mini Demographic and Health Survey (EMDHS). The Mini EDHS was a community-based cross-sectional study that covered all the administrative regions of Ethiopia. The data collection was conducted between March 21, 2019 and June 28, 2019. Both descriptive and analytic findings were produced. The overweight/obesity was measured by the weight-for-height (WFH) index, more than two standard deviations (+2 *SD*) above the median of the reference population based on the BMI *Z*-score. To identify significantly asso

**Results:**

A total of 5,164 under-five children were included in this study cited factors of overweight/obesity, a multilevel binary logistic regression model was fitted to account for the hierarchical nature of the data. Adjusted odds ratio (aOR) with a corresponding 95% confidence interval (*CI*) was reported to show the strength of association and statistical significance. The overall prevalence of overweight/obesity was 2.14% (95% *CI*: 1.74–2.53). The odds of overweight/obesity was higher among children aged <6 months (*aOR* = 5.19; 95% *CI*: 2.98–9.04), 6–24 months (*aOR* = 1.97; 95% *CI*: 1.18–3.29), delivered by cesarean section (*aOR* = 1.75; 95% *CI*: 1.84–3.65), living in Addis Ababa city (*aOR* = 2.16; 95% *CI*: 1.59–7.81), Oromia region (*aOR* = 1.93; 95% *CI*: 1.71–5.24), having mothers with the age 40–49 years (*aOR* = 3.91; 95% *CI*: 1.90–16.92), uses traditional contraceptive methods (*aOR* = 2.63; 95% *CI*: 1.66–10.47) and households headed by male (*aOR* = 1.71; 95% *CI*: 1.84–3.48).

**Conclusion:**

This study showed that the prevalence of overweight/obesity among under-five was low in Ethiopia. There were several factors that affect childhood overweight/obesity including child age, maternal age, mode of delivery, sex of head of household, contraception use, and geography of residence. Therefore, strategies to reduce childhood overweight and obesity should consider an identified multitude of contributing factors.

## Background

According to the World Health Organization (WHO), overweight and obesity are defined as abnormal or excessive fat accumulation that can impair health (1). In 2019 and 2020, an estimated 38.2 and 39 million children under the age of 5 years were overweight or obese, respectively (1). Although childhood overweight and obesity have been considered the problems of high-income countries, are now on the rise in low- and middle-income countries. While these countries continue to deal with the problems of infectious diseases and undernutrition, childhood overweight, and obesity are “double burdens” and the emerging public health challenges of the twenty-first century. Since 2000, the number of overweight children under the age of 5 has increased by nearly 24% in Africa (1, 2).

The number of overweight and obese children under the age of five has nearly doubled from 5.4 million in 1990 to 10.3 million in 2014, in Africa (3). According to 26 Demographic and Health Surveys carried out in SSA since 2010, overweight and obesity in the children within the age group of 0–59 months were reported 6.8% (4). A previous study suggested that childhood overweight and obesity in Sub-Saharan Africa (SSA) is likely to be worsened by the current transition in nutrition and physical activity that is characterized by increased use of energy-saving devices, and availability of cheap high-calorie-dense foods, and limited participation in physical activity generally (5).

In low-income Sub-Saharan countries including Ethiopia, childhood obesity is not yet perceived as an emerging health issue and receives little attention. According to the annual report of UNICEF in 2017, there was an overall increment of the prevalence of overweight among children from 1.7 to 3.6% in Ethiopia (6). According to 26 Demographic and Health Surveys carried out in SSA since 2010, it was reported 3% in Ethiopia (4). Although many studies have been conducted on the undernutrition of under-five children in Ethiopia, few studies reported the prevalence of childhood over-nutrition. A study conducted in Hawassa town reported the combined prevalence of childhood obesity and overweight which was 10.7% with 3.4% overweight, and 7.3% obesity. The combined prevalence was also 13.8% in Gondar town and 11.30% among children and adolescents in Ethiopia (7–9).

Childhood overweight and obesity are linked to more deaths than underweight and are associated with a higher chance of breathing difficulties, increased risk of fractures, hypertension, early markers of cardiovascular disease, insulin resistance, psychological effects, and adulthood obesity, premature death, and disability and resulting in an increased risk of non-communicable diseases and reproductive disorders later in their life (1, 10).

Previous studies reported many factors that were associated with overweight and obesity in under the age of five children. These include socioeconomic status, maternal education level, marital status, smoking during pregnancy, sex of the child, birth weight and the child's birth rank, residence, age of the child, body mass index (BMI) of parents, high dietary diversity, consumption of sweet food, and time spent in watching television >2 h/day (7, 11–13).

Despite the rising of childhood overweight and obesity in Ethiopia, there is a paucity of information with the robust statistical analysis given the clustering of data and hierarchical nature of the variables. This study applied a robust multilevel analysis considering the individual and community level factors contributing to childhood overweight/obesity. Therefore, this study aimed to assess the prevalence of overweight/obesity and determinants among under-five-aged children from the current Mini Ethiopian demographic and health survey data (MEDHS, 2019) by using multilevel analysis. Thus, this finding could be an input in the designing effective preventive strategies to alleviate the rising burden of early childhood overweight/obesity and its consequential morbidity and mortality in children and later adulthood life.

## Methods and materials

### Study setting, design, and population

This study used the 2019 Ethiopian Mini-EDHS data, which were collected by the Ethiopian Public Health Institute (EPHI), in partnership with the Central Statistical Agency (CSA) and the Federal Ministry of Health (FMOH). Ethiopia is located in North-eastern Africa between 3° and 15° North latitudes and 33° 48° and East longitudes. The country has eleven regional states and two city administrations (Addis Ababa and Dire Dawa) (13, 14). The survey was conducted in a nationally representative sample and provided estimates at national and regional levels for rural and urban areas (15).

A community-based cross-sectional study was conducted and data collection was done from March 21, 2019, to June 28, 2019. In this survey, 8,663 households were included and, data were collected from 5,753 children <60 months, 8,855 women of reproductive age (age from 15 to 49 years), and 40,659 household members (15).

A stratified two-stage cluster sampling technique was applied. In the first stage, a total of 305 enumeration areas (EAs) (93 in urban areas and 212 in rural areas) were selected with probability proportional to EA size (based on the 2019 EPHC frame). In the second stage, a fixed number of 30 households per cluster were selected with an equal probability of systematic selection from the newly created household listing. From all selected households, height and weight measurements were collected from children aged 0–59 months, and women aged 15–49 were interviewed using the Woman's Questionnaire (15).

### Data source

Before downloading the data, an approval letter was obtained from the DHS and then the dataset was downloaded from the DHS website (http://www.measuredhs.com). The kids' record dataset was used from the downloaded datasets. Anthropometric data of under-five children and various pertinent socio-demographic variables were extracted from the datasets. The nutritional status of under-five children was measured using the weight-for-height (WFH) index. The WFH index was later categorized into normal weight (above minus two standard deviations (−2 *SD*) and below plus two standard deviations (+2 *SD*) from the median of the reference population), severely wasted (below minus 3 (−3 SD) from the median reference population), moderately or severely wasted (below minus two standard deviations (−2 *SD*) from the median of the reference population), and overweight and obese (more than two standard deviations (+2 *SD*) above the median of the reference population) based on the BMI *Z*-score (1, 12, 16).

### Study variables

The outcome variable for this study was overweight/obesity in under-five children and it was coded as “1,” whereas those children who were categorized as underweight, and normal were coded as “0”. The independent variables were: Individual-level factors such as child age, child sex, birth order, twin child, delivered by cesarean section, mother's education status, current age, marital status, child ever born, age of mother at first birth, number of household members, sex of head of households, water source, toilet facility, wealth index, and contraceptive use. The community-level factors such as residence, region, and the altitude of clusters.

### Data management and statistical analysis

Before analysis, we cleaned, categorized, and recorded different variables using STATA version 14. A total of 5,164 under-five children were included in this analysis after excluding those children whose BMI *z*-scores were missing or were recorded as out of plausible limits” or “Flagged cases,” as their values were unusable. These data were recorded in the database under special codes which corresponded either to responses that were considered inconsistent with other responses in the questionnaire and thought to be probably an error or to responses whose value was “Don't know” (12, 16).

Both descriptive statistics such as frequencies and proportions and analytic were computed. A weighted analysis was done to compensate for the unequal probability of selection between the strata due to the non-proportional allocation of samples to different regions, places of residence, and non-response rate among study participants (15). Since demographic and health survey (DHS) data had a clustering and hierarchical nature. The individual-level factors (level 1) were nested within communities (level 2). A two-level mixed-effect logistic regression model was fitted to estimate both the independent (fixed) effect of the explanatory variables and the community-level (random) effect on our dependent variable childhood overweight/obesity (14). The rate of childhood overweight/obesity varies from cluster to cluster; a cluster-level random intercept was introduced in the mixed logit model. The within-cluster correlation was measured using intra-cluster correlation (ICC) which was 21.6 % which indicated that the variations of overweight/obesity in under-five children were attributable to the difference at cluster level factors. The likelihood ratio test was applied to test the significance of the variance of random intercept. Bivariable multilevel logistic regression analysis was performed and those variables with *p*-value <0.25 were considered for the multivariable analysis (17). Adjusted odds ratio with the corresponding 95% confidence level was computed and reported to show the strength of the association and its significance. Variables having a *p*-value <0.05 were considered as having a significant association with the outcome variable. The deviance information criterion (DIC) was used to check the model goodness of fit.

Regarding the model building, four models were fitted. The first model was the null model (Model 0) containing no independent variables which were used to check the variability of overweight/obesity in the community and provide evidence to assess random effect using the ICC. The ICC in the null model was 0.21, which means about 21.60% of the variations of overweight/obesity in the under-five-aged children were attributable to the difference at cluster level or community-level factors. The second model (Model I) contains individual-level variables and the third model (Model II) contains community-level variables. Both individual and community level variables were fitted simultaneously with the outcome variable in the fourth model (Model III). The model comparison was done using deviance and the fourth model with the lowest deviance (831.10) was selected as the best-fitted model (Table 1).

**Table 1:** Random effect and model comparison for factors associated with childhood overweight/obesity.

**Measures**	**Null model**	**Model I**	**Model II**	**Model III**
Intraclass correlation coefficient (ICC)	21.60 %	18.54 %	16.35 %	15.15 %
Intercept	0.90(0.42–1.92)	0.75 (0.32–1.73)	0.64 (0.26–1.62)	0.59 (0.21–1.63)
Akaike information criterion (AIC)	913.24	893.77	915.84	903.10
**Model fitness**				
Log-likelihood	−454.62	−422.89	−443.92	−415.55
Deviance	909.24	845.78	887.84	831.10

## Results

### Socio-demographic characteristics

A total of 5,164 under-five children were included in the analysis. About 57.54% of children were found between the ages of 25–59 months and in a balanced male-to-female proportion, where 50.87% were boys. A majority 75.07% were rural residents and 39.82% were from the Oromia region. Only 2.31 and 5.16% of children were twin and delivered by cesarean section, respectively (Table 2).

**Table 2:** Socio-demographic characteristics of under-five children in Ethiopia, Ethiopia Mini Demographic and Health Survey (EMDHS) 2019.

**Variables**	**Un-weighted frequency (%)**	**^a^Weighted frequency (%)**
**Age of child in months [mean age = 28.94 ± 17.19 (SD)]**		
<6 months	566 (10.96)	523.37 (10.41)
6–24 months	1,613 (31.24)	1,612.23 (32.05)
25–59 months	2,985 (57.80)	2,894.16 (57.54)
**Sex of child**		
Male	2,636 (51.05)	2,558.82 (50.87)
Female	2,528 (48.95)	2,470.94 (49.13)
**Residence**		
Urban	1,182 (22.89)	1,253.89 (24.93)
Rural	3,982 (77.11)	3,775.86 (75.07)
**Region**		
Tigray	432 (8.37)	351.86 (7.00)
Afar	585 (11.33)	76.52 (1.52)
Amhara	470 (9.10)	965.85 (19.20)
Oromia	649 (12.57)	2,002.89 (39.82)
Somalia	546 (10.57)	346.46 (6.89)
Benishangul	468 (9.06)	60.25 (1.20)
SNNPR	609 (11.79)	1,025.21 (20.38)
Gambella	392 (7.59)	21.64 (0.43)
Harari	397 (7.69)	14.66 (0.29)
Addis Ababa	258 (5.00)	138.21 (2.75)
Diredawa	358 (6.93)	26.22 (0.52)
**Birth order**		
One	1,081 (20.93)	1,065.82 (21.19)
Two to three	1,729 (33.48)	1,613.59 (32.08)
Four to six	1,643 (31.82)	1,589.09 (31.59)
Seven and above	711 (13.77)	761.26 (15.14)
**Child is twin**		
Yes	118 (2.29)	116.16 (2.31)
No	5,046 (97.71)	4,913.60 (97.69)
**Delivered by cesarean section**		
Yes	304 (5.89)	259.59 (5.16)
No	4,860 (94.11)	4,770.17 (94.84)
Cluster altitude (in meters)	(Mean = 1,537.51 ± (686.91 SD), min = 246, max = 3,246	

a The sampling weights were applied to obtain the weighted frequency.

### Household and maternal characteristics

From mothers in the reproductive age, 31.57% were in the age category of 25–29 years, the least 1.81% were in the age category of 45–49 years. At the first birth, about 42.07% of women were under the age of 18 years. Regarding the education status of mothers, the majority 53.92% had no formal education followed by primary, secondary, and above 35.19, 7.29, and 3.60%, respectively. About 86.98% of heads of households were male and the majority 23.57% of households was in the poorest wealth index rank (Table 3).

**Table 3:** Household and maternal characteristics of under-five children mini EDHS 2019.

**Variables**	**Un-weighted** **frequency (%)**	**^a^Weighted** **frequency (%)**
**Maternal age**		
15–19 years	238 (4.61)	218.79 (4.35)
20–24 years	996 (19.29)	926.72 (18.42)
25–29 years	1,694 (32.80)	1,587.91 (31.57)
30–34 years	1,145 (22.17)	1,121.57 (22.30)
35–39 years	701 (13.57)	756.59 (15.04)
40–44 years	305 (5.91)	327.05 (6.50)
45–49 years	85 (1.65)	91.13 (1.81)
**Age at first birth**		
Under 18 years	2,234 (43.26)	2,116.18 (42.07)
18 and above	2,930 (56.74)	2,913.57 (57.93)
**Maternal educational level**		
No education	2,855 (55.29)	2,712.15 (53.92)
Primary	1,602 (31,02)	1,769.81 (35.19)
Secondary	434 (8.40)	366.57 (7.29)
Above	273 (5.29)	181.23 (3.60)
**Religion**		
Orthodox	1,486 (28.78)	1,716.83 (34.13)
Muslims	2,619 (50.72)	1,843.97 (36.66)
Protestant	956 (18.51)	1,370.39 (27.25)
Catholic	29 (0.56)	17.15 (0.34)
Others	74 (1.43)	81.41 (1.62)
**Marital status**		
Never in union	27 (0.52)	17.95 (0.36)
Married	4,839 (93.71)	4,744.84 (94.34)
Living with partners	34 (0.66)	33.90 (0.67)
Widowed	54 (1.05)	58.17 (1.16)
Divorced	143 (2.77)	121.42 (2.41)
Separated	67 (1.30)	53.47 (1.06)
**Sex of head of household**		
Male	4,150 (80.36)	4,374.98 (86.98)
Female	1,014 (19.64)	654.77 (13.02)
**Child ever born**		
<4	2,528 (48.95)	2,445.47 (48.62)
Four and above	2,636 (51.05)	2,584.28 (51.38)
**Wealth index**		
Poorest	1,755 (33.99)	1,185.73 (23.57)
Poorer	904 (17.51)	1,099.18 (21.85)
Middle	716 (13.87)	936.84 (18.63)
Richer	667 (12.92)	878.89 (17.47)
Richest	1,122 (21.73)	929.11 (18.47)
**Contraceptive use**		
Using modern methods	1,682 (32.57)	2,054.61 (40.85)
Using traditional methods	44 (0.85)	36.81 (0.73)
Do not intend to use	3,438 (66.58)	2,938.33 (58.42)
**Source of drinking water**		
Safe	2,318 (44.89)	2,199.04 (43.72)
Not safe	2,846 (55.11)	2,830.71 (56.28)
**Toilet facility**		
Yes	3,025 (58.58)	3,434.29 (68.28)
No	2,139 (41.42)	1,595.46 (31.72)
**Family size**		
<5	1,383 (26.78)	1,427.81 (28.39)
Five and above	3,781 (73.22)	3,601.94 (71.61)

a The sampling weights were applied to obtain the weighted frequency.

### Prevalence of overweight/obesity among under-five children in Ethiopia

**Figure 1:**
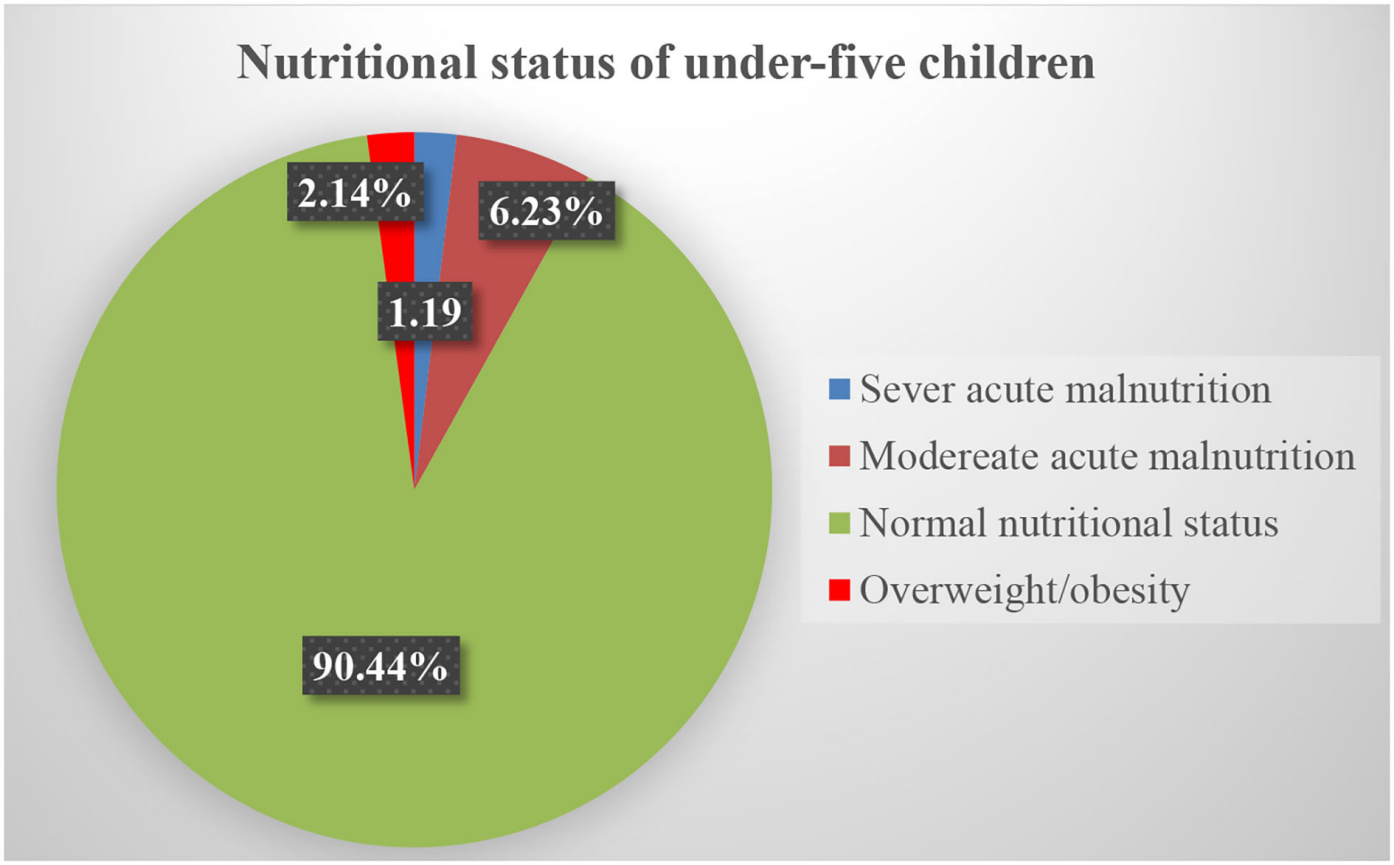
Nutritional status in under-five aged children in Ethiopia: Data from Ethiopia Mini Demographic and Health Survey (EMDHS 2019).

Among all under-five children, the weighted analysis indicated that 2.14% (95% *CI*; 1.74–2.53) children were overweight/obese. The majority (90.44%) of under-five children were in the normal range of weight for height *SD* scores (*Z*-scores), 7.42% underweight, and 2.14% overweight/obesity (Figure 1).

### Factors associated with overweight/obesity

In the bivariable multilevel binary logistic regression analysis, child age, child sex, current maternal age, maternal age at first birth, maternal educational level, sex of household head, child ever born, household wealth index, contraceptive use, child delivered by cesarean section, household toilet facility, residence, region, and altitude of the cluster were associated to the childhood overweight/obesity (*p*-value <0.25). In the multivariable multilevel binary logistic regression analysis, child age, maternal age, sex of household head, contraceptive use, child delivered by cesarean section, and region were significantly associated with under-five children overweight/obesity (*p*-value <0.05) (Table 4).

**Table 4:** Multilevel binary logistic regression analysis for factors associated with overweight/obesity in under-five children, in Ethiopia.

**Variables**	**cOR (95% CI)**	**Model-I (aOR)** **(95% CI)**	**Model-II (aOR)** **(95% CI)**	**Model-III (aOR)** **(95% CI)**
**Child age**				
<6 months 6–24 months 25–59 months	5.06 (2.96–8.63) 1.91 (1.16–3.15) 1.00	5.10 (2.93–8.88) 1.94 (1.16–3.23) 1.00		5.19 (2.98–9.04)^*^ 1.97 (1.18–3.29)^*^ 1.00
**Child sex**				
Male Female	1.00 1.33 (0.87–2.03)	1.00 1.25(0.81–1.93)		1.00 1.25 (0.81–1.92)
**Maternal age**				
–19 years 20–24 years 25–29 years 30–34 years 35–39 years 40–49 years	1.00 0.90 (0.29–2.79) 1.11 (0.38–3.23) 0.99 (0.33–2.99) 0.45 (0.12–1.64) 1.57 (0.47–5.21)	1.00 1.14 (0.35–3.69) 1.82 (0.57–5.85) 1.89 (0.54–6.63) 0.98 (0.22–4.26) 4.02(0.94–17.17)		1.00 1.17 (0.36–3.83) 1.81 (0.56–5.85) 1.91 (0.54–6.72) 0.95 (0.22–4.20) 3.91 (1.90–16.92)^*^
**Maternal age at first birth**				
Under 18 years 18 and above	1.00 1.44 (0.92–2.25)	1.00 0.98 (0.58–1.65)		1.00 0.95 (0.56–1.61)
**Maternal educational status**				
No education Primary Secondary Above	1.00 1.43 (0.87–2.35) 2.32 (1.18–4.56) 1.79 (0.77–4.19)	1.00 1.22 (0.69–2.16) 1.71 (0.76–3.84) 1.10 (0.40–3.03)		1.00 1.14 (0.64–2.04) 1.59 (0.69–3.66) 1.16 (0.42–3.22)
**Sex of head of household**				
Male Female	1.86 (0.94–3.68) 1.00	1.82 (0.90–3.68) 1.00		1.71 (1.84–3.48)^*^ 1.00
**Child ever born**				
Less than four Four and above	1.55 (1.01–2.40) 1.00	1.49 (0.78–2.83) 1.00		0.66 (0.34–1.28) 1.00
**Wealth index**				
Poorest Poorer Middle Richer Richest	1.00 1.49 (0.77–2.86) 0.76 (0.31–1.85) 1.68 (0.82–3.45) 1.95 (1.05–3.64)	1.00 1.21 (0.59–2.48) 0.57 (0.22–1.49) 1.14 (0.49–2.66) 1.11 (0.47–2.64)		1.00 1.05 (0.49–2.20) 0.48 (0.18–1.28) 0.93 (0.39–2.22) 0.75 (0.24–2.28)
**Contraceptive use**				
Modern methods Traditional methods Do not intend to use	1.00 3.46(0.92–12.97) 0.89 (0.57–1.42)	3.11(0.77–12.52) 1.01 (0.61–1.66)		1.00 2.63 (1.66–10.47)^*^ 1.05 (0.64–1.73)
**Child delivered by caesarean section**				
Yes No	2.43 (1.26–4.69) 1.00	1.77 (0.86–3.65) 1.00		1.75 (1.84–3.65)^*^ 1.00
**Toilet facility available**				
Yes No	1.53 (0.94–2.51) 1.00	0.82 (0.45–1.51) 1.00		0.86 (0.45–1.63) 1.00
**Residence**				
Urban Rural	1.56 (0.91–2.66) 1.00		1.11 (0.57–2.14) 1.00	0.97 (0.40–2.35) 1.00
**Region**				
Tigray	1.00 0.63		1.00 0.68 (0.17–2.70)	1.00
Afar	(0.20–2.00) 0.44		0.43 (0.11–1.63) 1.79	0.77 (0.18–3.25)
Amhara	(0.12–1.63)		(0.69–4.71) 0.38	0.49 (0.13–1.89)
Oromia	1.79 (0.69–4.69)		(0.09–1.66) 0.68	1.93 (1.71–5.24)^*^
Somalia	0.35 (0.09–1.35)		(0.19–2.43) 0.89	0.49 (0.10–2.31)
Benishangul	0.65 (0.19–2.18)		(0.31–2.57) 0.61	0.69 (0.18–2.65)
SNNPR	0.88 (0.31–2.56)		(0.13–2.85) 1.57	0.89 (0.29–2.76)
Gambella	0.56 (0.15–2.12)		(0.53–4.66) 2.09	0.61 (0.13–2.96)
Harari	1.62 (0.56–4.72)		(0.59–7.37) 0.76	1.69 (0.55–5.18)
Addis Ababa	2.35 (0.81–6.89)		(0.20–2.84)	2.16 (1.59–7.81)^*^
Diredawa	0.75 (0.21–2.63)			0.74 (0.19–2.89)
**Cluster altitude**	1.01(1.01–1.02)		1.001(0.99–1.01)	1.00 (0.99–1.001)

* P-value <0.05, **Model I**: individual-level factors **Model II**: community-level factors **Model III**: Both community and individual-level factors.

Children in the age category of fewer than 6 months were 5.19 times more likely to be overweight/obese as compared to those children 25–59 months (*aOR* = 5.19; 95% *CI*: 2.98–9.04). Similarly, children in the age group of 6–24 months were 1.97 times more likely to be overweight/obese as compared to those children 25–59 months (*aOR* = 1.97; 95% *CI*: 1.18–3.29). Maternal age was significantly associated with the nutritional status of under-five children. Children whose mothers age 40–49 years were 3.91 times more likely to be overweight/obese as compared to the children with maternal age from 15 to 19 years (*aOR* = 3.91; 95% *CI*: 1.90–16.92). The odds of overweight/obesity were higher in those children whose household head was male. Those children in the household headed by men had 1.71 times odds of overweight/obesity when compared to those children in the household headed by a female (*aOR* = 1.71; 95% *CI*: 1.84–3.48). Contraceptive use was another factor significantly associated with childhood overweight and obesity. Under-five children whose mothers used traditional contraceptive methods were 2.63 times more likely to have overweight/obesity when compared to those children whose mothers used modern contraceptive methods (*aOR* = 2.63; 95% *CI*: 1.66–10.47). Furthermore, children delivered by cesarean section were 1.75 times more likely to be overweight/obese as compared to their counterparts (*aOR* = 1.75; 95% *CI*: 1.84–3.65). The risk of overweight/obesity was 2.16 times higher in children's lives in Addis Ababa administrative city as compared to children in the Tigray region (*aOR* = 2.16; 95% *CI*: 1.59–7.81). Likewise, under-five children in the Oromia region were 1.93 times at higher risk of overweight/obesity as compared to children who live in the Tigray region (*aOR* = 1.93; 95% *CI*: 1.71–5.24) (Table 4).

## Discussion

In Ethiopia, childhood overweight/obesity is not yet perceived as an emerging health issue. According to United Nations International Children's Emergency Fund (UNICEF) annual report, there is an overall increment in the prevalence of overweight/obesity among children from 1.7 to 3.6% in Ethiopia (18). Hence, this study assessed the prevalence of overweight/obesity and associated factors among under-five children in Ethiopia. The prevalence of overweight/obesity in this study was 2.14% (95% *CI*: 1.74–2.53). The factors associated with childhood overweight/obesity were: age of children, living regions, delivery by cesarean section, age of mothers, contraceptive use of mothers, and sex of head of households.

The magnitude of overweight/obesity observed in this study is consistent with the study done in Bahir Dar town (6.9 %) (19). The current finding was lower than the findings of the study in different settings; 13.8% in Gondar (7), and the combined prevalence was 10.7% in Hawassa with 7.3% overweight and 3.4% obesity (9).

The prevalence of overweight/obesity in this study was also lower than in a study conducted in Cameroon which was 8% (11), in Kenya 20.3% (20), in Sub-Saharan Africa 6.8% (4) in Nigeria 23.6% (21). A study done in Iran reported that 11.8% overweight and 15% obese (22), in urban and rural Vietnam, 21.1 and 7.6%, respectively (23). In Malawi, the combined prevalence of childhood overweight and obesity was 14.5% with the 8.7% overweight and 5.8% obesity (4); in Mozambique 11.9% (7.7% overweight and 4.2% obesity) (4), and in Brazil, the overweight was 9.7% (24). In Iraq, the combined prevalence of overweight and obesity was 11.2% (7.6% overweight and 3.6% obesity) (25), in Iran 35.7% (12% overweight and 23.7% obesity) (22), and in Brazil, it was 21.9% with the 14.4% overweight and 7.5% obese (26). The finding of the present study was also lower than the global prevalence of overweight/obesity which was 7% (27). The observed difference might be due to the variations in socio-cultural and socio-economic status in dietary intake, lifestyle, and study time.

In the current study, the age of the child was significantly associated with childhood overweight/obesity. The odds of overweight and obesity were 5.19 times higher among children whose age group was fewer than 6 months as compared to those children in the age group of 25–59. Similarly, children in the age category of 6–24 months had 1.97 times higher odds of overweight and obesity when compared to those children in the age group of 25–59 months. This finding was in agreement with the study conducted in Gondar, Hawassa, Ethiopia, and Cameroon (7, 9, 11, 12). In these study findings, younger children had a higher risk of becoming overweight or obese than their older comparative age groups. The possible reason might be that when the age of the child increases, the chance of joining the school would increase and this may attribute to physical activity, which would lead to increased metabolic activity and energy requirements. The other possible explanation may be during the early childhood period physiologically the percentage of body fat decreases and muscle tissue increases and children get thinner. This study report suggests that age-specific nutritional counseling programs and strategies during childhood are necessary.

The present study showed that maternal age was significantly associated with the overweight and obesity of under-five children. The odds of under-five children overweight and obesity was 3.91 times higher among children whose maternal age was 40–49 years as compared to those children whose maternal age was from 15 to 19 years. The possible explanation might be as the age of mothers increase, the body mass index (BMI) also does increase. Previous studies reported that there is a positive association between the BMI of mothers and the overweight and obesity of children (28). For this association, the following reasons were suggested: these include inheritance of genes that enhance susceptibility, and the behaviors of poor eating habits (29, 30).

This study revealed that the risk of overweight and obesity was higher in those children whose households were headed by men. Those children in the household headed by men had 1.71 times higher odds of overweight and obesity when compared to those children in the household headed by a female. This might be because of the food security status of the household. Pooled estimate from systematic-review and meta-analysis in Ethiopia indicated that the households headed by men had two-fold times higher odds of food security when compared to the female-headed households (31). Hence, those households headed by men might encourage their children to take energy-dense foods that are high in fat and sugars and change in modes of transportation, which increases sedentary life this leads to physical inactivity.

Contraceptive use was another factor significantly associated with childhood overweight and obesity. Under-five children whose mothers used traditional contraceptive methods were 2.63 times more likely to have overweight and obesity when compared to those children whose mothers used modern contraceptive methods. The possible reason might be those mothers who use traditional contraceptive methods may have low awareness about the effects of energy-dense foods that are high in fat and sugars, and physical inactivity due to sedentary lifestyles. However, the comparative category of mothers of under-five children who use modern contraceptive methods may have good awareness because of the variety of health education during attending the family planning schedule in health facilities.

Furthermore, children delivered by cesarean section were 1.75 times more likely to be overweight and obese as compared to their counterparts. The association between cesarean delivery and childhood overweight and obesity is not clear. However, the possible reason can be the weight of the child at birth. Previous studies showed that a child overweight at birth was a risk for cesarean delivery as well as for overweight and obesity in later life (32, 33). The other suggested possible mechanism was the hygiene hypothesis (34, 35). In this hypothesis, cesarean delivery deprives the chance for the newborn baby to be exposed to maternal vaginal feces, the bacteria from which are a major source for the intestinal bacteria of the newborn. As a result, as compared with those born vaginally, newborns delivered by cesarean section had fewer intestinal Bifidobacteria and Bacteroides both of which were reported to be protective factors against later obesity (36, 37). The association was also supported by indirect epidemiological evidence, in addition to hygiene theory (38). Cesarean delivery was associated with a lower umbilical leptin concentration and it reduces the rate of early breastfeeding both of which were reported to be associated with an increased risk of later overweight and obesity (39–42).

The risk of overweight and obesity was 2.16 times higher in children living in Addis Ababa administrative city as compared to children in the Tigray region. Likewise, under-five children living in the Oromia region were 1.93 times at higher risk of overweight and obesity as compared to children living in the Tigray region. This variation might be due to differences in the habit of food eating and sedentary lifestyle. Because of the better socio-economic status of the population, and the better access to high-calorie diets in Addis Ababa compared to the Tigray regions of the country.

## Strengths and limitations of the study

The study used a large survey and nationally representative data including regional variation and factors at individual and community levels. However, there were some limitations to this study. The cross-sectional nature of the data prevents causality from being inferred between the independent and dependent variables. This study only focused on the specific factors of overweight and obesity but did not include missed variables such as dietary intake, feeding habits of the children, maternal nutritional status, and the weight of the child at birth. These might be the residual confounding factors. This study assessed the information from the 5 years before the survey period; it might be prone to recall bias, particularly for age or other retrospective data relying on memory of past events.

## Conclusion

This study showed that the prevalence of overweight/obesity among under-five was low in Ethiopia. Child factors, maternal-related factors, healthcare utilization, and geography of residence commonly contributed to the prevalence of overweight/obesity. Therefore, giving emphasis to children with these identified factors and formulating preventive programs and policies during children's early years are highly recommended.

## Data availability statement

Publicly available datasets were analyzed in this study. This data can be found here: http://www.measuredhs.com.

## Ethics statement

Ethical review and approval was not required for the study on human participants in accordance with the local legislation and institutional requirements. Written informed consent to participate in this study was provided by the participants' legal guardian/next of kin.

## Author contributions

MG involved in the conception, acquisition of data, analysis, interpretation, and drafting of the manuscript. MM, SH, HA, AA, and BW involved in the interpretation and drafting of the manuscript. All authors read and approved the final manuscript and agreed to be personally accountable for the authors' contributions and to ensure that questions related to the accuracy or integrity of any part of this study.

## Conflict of interest

The authors declare that the research was conducted in the absence of any commercial or financial relationships that could be construed as a potential conflict of interest.

## Publisher's note

All claims expressed in this article are solely those of the authors and do not necessarily represent those of their affiliated organizations, or those of the publisher, the editors and the reviewers. Any product that may be evaluated in this article, or claim that may be made by its manufacturer, is not guaranteed or endorsed by the publisher.

## Abbreviations

aOR: adjusted odds ratio
AIC: Akaike information criterion
BMI: body mass index
*CI*: confidence interval
cOR: crude odds ratio
CSA: central statistics agency
EA: enumeration areas
EMDHS: Ethiopian mini demographic and health survey
EPHI: Ethiopian public health institute
FMoH: Federal ministry of health
ICC: Intra-class correlation coefficient
PHC: Population and housing census
SSA: Sub-Saharan Africa.
